# The Selective Impairment of the Phonological Output Buffer: Evidence From a Chinese Patient

**DOI:** 10.1155/2005/647871

**Published:** 2006-01-09

**Authors:** Hua Shu, Hanzhong Xiong, Zaizhu Han, Yanchao Bi, Xiaoli Bai

**Affiliations:** ^1^State Key Laboratory of Cognitive Neuroscience and LearningBeijing Normal UniversityChina; ^2^Hubei Normal UniversityChina; ^3^Harvard UniversityUSA; ^4^Friendship Hospital of BeijingChina

**Keywords:** Phonological output buffer, Chinese, oral production

## Abstract

We present a Chinese-speaking patient, SJ, who makes phonological errors across all tasks involving oral production. Detailed analyses of the errors across different tasks reveal that the patterns are very similar for reading, oral picture naming, and repetition tasks, which are also comparable to the error patterns of the phonological buffer deficit cases reported in the literature. The nature of the errors invites us to conclude that the patient's phonological output buffer is selectively impaired. Different from previously reported cases, SJ's deficits in oral production tasks are not accompanied by a similar impairment of writing performance. We argue that this dissociation is evidence that the phonological output buffer is not involved in writing Chinese words. Furthermore, the majority of SJ's errors occur at the onset of a syllable, indicating that the buffer has a structure that makes the onset more prone to impairment.

## 1. Introduction

The concept of a phonological output buffer has a long history in the context of short-term (working) memory studies (e.g. [1,7,19]), where it is proposed as a short-term auditory-verbal store linking to the articulatory loop system. The postulation of such a buffer system in language processing is theoretically well motivated. In general, when the "unit" of information that is output from one representation is larger than what the next representation can take as "input" for further processing, it is reasonable to assume the existence of a buffer to hold the to-be-processed units temporarily. An intuitive example can be found in the process of writing words. In word writing, after an output lexical node is selected, it activates its graphemic representation. The activated graphemes may be held in a (graphemic) buffer before the final letter-by-letter output stage (e.g. [6]).

Similarly, in oral sentence production, the phonological contents of more than one word might be activated, whereas the articulation system operates in a phoneme-by-phoneme fashion (e.g. [16]). A phonological output buffer can hold the to-be-articulated phonological information. The empirical justification for the exact functional role of such a phonological buffer in word processing has come mainly from patients who are assumed to have selective phonological buffer impairment [2,3,5,12,15]. Although to our knowledge, there are only five well-documented cases with selective phonological output buffer impairment, these cases have important theoretical implications for the mechanisms of reading, writing, and oral naming of words

ISSN 0953-4180/05/$17.00 © 2005 – IOS Press and the authors. All rights reserved and nonwords. It was proposed that impairment of such a buffer will affect various processes, including repetition, reading, oral picture naming, and the writing of nonwords and maybe words. In this article, we report a new Chinese-speaking patient with selective phonological output buffer impairment, with a special focus on examining the hypothesis that the phonological buffer is involved not only in oral production modalities but in writing as well.

Caramazza, Miceli, and Villa [5] reported the first case that was identified as having a specific deficit with respect to the phonological (output) buffer. Their patient, IGR, made errors in reading (aloud), repetition, and writing-to-dictation of nonwords. The errors he made were comparable across these three tasks both by quality and by quantity. All of the errors the patient made were phonologically related to the target, the majority of which were single phoneme errors. Across all three tasks, phoneme substitution errors were the most frequent type, the erroneous responses were often of the same manner of articulation as the target and the most frequent position of errors was also similar. At least three other cases with similar overall performance were reported later: GC [12], MV [3] and RR [2].

On the basis of association logic, Caramazza et al. [5] proposed that the deficit of the same cognitive component underlies the failures in tasks involving different input and output modalities. Functional analyses of the three kinds of tasks and the phonological nature of the errors suggest that the phonological output buffer is the locus of the deficits. In both oral reading and repetition of nonwords, the phonological buffer has to be involved prior to the articulatory system. In nonword writing, the phonological buffer is necessary to hold phonemes before they are converted into graphemes for written output. The fact that IGR did not make paraphasia errors in spontaneous speech or real word tasks-reading, repetition, and writing-led Caramazza et al. [5] to propose that the oral and written production of real words can bypass the phonological buffer. The written production of words can go from the orthographic output lexicon to the graphemic buffer (the classical "lexical route" of writing [14]), the orthographic lexicon being activated either directly from the semantic system or through the phonological lexicon. The oral production of words can be achieved by a direct link between the phonological output lexicon and the "lexical articulatory system."

Bub et al. [3] proposed a different explanation for the dissociation between the performance on word tasks and nonword tasks in their patient, which had a similar pattern to IGR's. They adopted the idea of "resource artifact" [13] and argued that the sparing of words is not because the phonological buffer is functionally irrelevant in word processing. Both word and nonword processing involve the phonological buffer. Words, however, have advantages over nonwords because patients can use information from the lexical representation to "compensate" for imperfect information in the buffer for words. If the deficit of the phonological buffer is mild enough, the impact on word processing may remain unseen, with the nonword processing difficulty present.

Bub et al.'s notion [3], although vague, gained support from a case, LT, reported by Shallice, Rumiati, and Zadini [15]. Although LT's impaired performance on nonwords is more severe in quantity than that of IGR, it is virtually equivalent in quality. Critically, LT's performance on word processing tasks (reading, repetition, and writing) exhibited the same patterns as IGR's, although they were less severe. The association between word and nonword processing led Shallice et al. [15] to argue that "whatever impairment affects the processing for nonwords also affects the processing for words."

To summarize, it has been repeatedly reported that patients appearing to experience phonological output buffer deficits show nearly equivalent patterns of errors in reading, repetition, and writing of nonwords (IGR, MV, RR, GC, LT). There is also a patient (LT) showing associated patterns on the same tasks with words. To explain the association across the three tasks and the two types of stimuli, it was proposed [3,15] that the phonological output buffer is a common processing component in all of these tasks with both words and nonwords, and it is the locus of the associated impairments.

This account is consistent with observed associations *prima facie*, but is it theoretically justified? As Caramazza et al. [5] suggested, "The role of a buffer is to hold temporary representations that do not correspond to the units of analysis at some point in the flow of information processing." Consistent with this description is the view that the oral production of words or nonwords requires a buffer to hold phonological representations before they are converted into the articulatory program, if one were to assume that the articulatory program operates over units that are larger than lexical phonological representations (for words) or any activated phonological contents (for nonwords). Because this buffer is assumed to come later in the process than the phonological lexical level, the error types should be independent of the kinds of the oral production tasks (reading or repetition).

However, it is far from straightforward to assume the same functional role of the phonological buffer in written production as in oral production. It has been established that there are at least two routes for writing: lexical and nonlexical (e.g. [11,14,21]). In the lexical route, going through the lexical orthographic representation of words, the activated lexical orthographic representation can be put directly into the graphemic buffer for written production. The other route, the nonlexical or sublexical route, is a path of generating the graphemic information from the auditory input through the sound-to-phoneme conversion. In this nonlexical conversion process, it is natural to assume that the to-be-converted phonological information is held in the phonological output buffer. However, a functional role of the phonological output buffer in the lexical route of writing is hardly necessary.

If we assume that there is a lexical route for writing that bypasses the phonological output buffer, how can we explain the fact that LT made errors in the same fashion on the word writing task as on other oral production tasks and on nonword processing tasks? One way is to assume that LT was also impaired somewhere along the lexical route for writing. This lexical deficit occurred in such a fashion that any possible lexical effect would be shadowed by the phonological buffer deficit. According to this account, we should see patients with an impaired phonological output buffer but an intact lexical route for writing who show normal performance in writing real words but phonological errors in oral production tasks (repetition, reading, oral naming) of both real words and nonwords.

Alternatively, one might assume that in writing to dictation, at least in transparent languages with highly regular sound-to-grapheme correspondence, the nonlexical route is much more powerful than the lexical route. In most cases, the graphemic information activated through the lexical route is not strong enough to compensate for the imperfect graphemic information converted from the impoverished phonological output buffer. This could explain why LT's performance on the word writing-to-dictation task was not better or qualitatively different from his performance on other oral production tasks. Only when the nonlexical route is severely impaired would the lexical route be relied on to write, as in "phonological disgraphia" patients [14].

In other words, the association between oral production and written production in phonological output buffer deficit patients is not purely accidental but could be a function of the phoneme-grapheme-regularity of the language. It is more likely to occur in a transparent language, compared to languages with opaque sound-to-grapheme correspondence, such as English or Chinese. Finally, it is possible the phonological output buffer is not completely absent in the lexical route of writing: some refreshing mechanism of the graphemic buffer might be phonological in nature and involve the phonological output buffer. Were this final hypothesis true, the association of phonological errors in word writing and the phonological buffer deficit would not be accidental or a function of the transparency of the language but, rather, a universal phenomenon.

Chinese is a logographic language where the phonology and orthography do not correspond on the segmental level but, instead, are syllable based. The basic writing units are characters that are composed of particular strokes structured in different spatial organizations. The basic phonological units are syllables containing onsets, rimes, and tones. It is often assumed that there is no nonlexical route for Chinese character writing that is comparable to that in alphabetic languages, and the character writing process can be accomplished either through the semantic system or a nonsemantic route might be mediated by the phonological lexicon (e.g. [8, 9,22]).

The Chinese speaking case we present here, SJ, makes phonological errors across all tasks involving oral production, including reading, repetition of words and nonwords, and oral picture naming. Detailed analyses of the errors across different tasks reveal that the error patterns are very similar for reading, oral picture naming, and repetition tasks, which are also comparable to the error patterns of the phonological buffer deficit cases reported in the literature. The nature of the errors invites us to speculate that the patient's phonological output buffer is impaired.

Therefore, one primary aim of the article is to document converging cases with such a deficit. We also focus on the issues of the functional role of the phonological output buffer in different modalities by comparing SJ's performance on different tasks and the nature of his errors. It is hoped that such a comparison will provide insights into the functional role of the phonological buffer in writing.

Recall that to account for the association between the phonological output buffer impairment and the writing deficit in LT, we mentioned three possibilities: 1) that LT also has a deficit in the lexical route of writing; 2) that the nonlexical route of writing is more dominant for transparent languages like Italian; and 3) that the phonological output buffer is involved in some refreshing mechanism of the graphemic buffer and, therefore, cannot be completely bypassed even in the lexical route of writing. Assuming that SJ is *selectively* impaired with the phonological buffer, the first two possibilities predict that SJ's word writing will not be similar to his oral production performance, given that in Chinese the nonlexical route of writing is not dominant, if it exists at all. The third hypothesis, however, predicts that SJ's writing will be affected by the phonological output buffer impairment as well. SJ's performances across various modalities were compared to examine the role of the phonological output buffer in the language processes.

A patient with specific phonological buffer impairment might also inform us about the structural characteristics of the buffer. By analyzing the principles that the errors exhibit in selective phonological output buffer deficit patients, one may understand in more detail how the phonological information is organized in the buffer. For instance, IGR's [5] erroneous phonemes were usually of the same manner of articulation as the target phonemes. Therefore, the phonological output buffer has to be structured in a way such that the manner of articulation can be less impaired than the knowledge about other phonological dimensions. SJ's erroneous patterns will also be analyzed along the phonological features of Mandarin to gather more information about the structure of the phonological buffer in that language.

We present the case study by addressing the following questions: 1) What are SJ's error patterns across different tasks? 2) What factors affect the likelihood of making errors? and 3) What kinds of characteristics do the errors have? Data from these empirical findings will be analyzed around the following theoretical issues: whether impairment of the phonological output buffer best captures SJ's behavior pattern; whether the phonological output buffer is involved in various language processes (e.g., reading, repetition, and writing); and what the structural characteristics of the phonological buffer are for Chinese speakers.

## 2. Methods and results

### 2.1. Case background

SJ was a 50-year-old, right-handed male with a college education. He worked as a university professor, having normal language capacity premorbidly. In March 2003, he was hospitalized due to a brain abnormality. The brain CT showed no obvious lesion, but the magnetic resonance images (MRIs) revealed ischemic damage in the left parietal lobe (Fig. 1). His spontaneous speech was relatively fluent, grammatically well constructed, and paraphasic errors were prevalent. SJ was aware of his errors and frequently attempted to correct them. He was diagnosed as a conduction aphasic. The data reported in this paper were collected during March 2003 and June 2003.

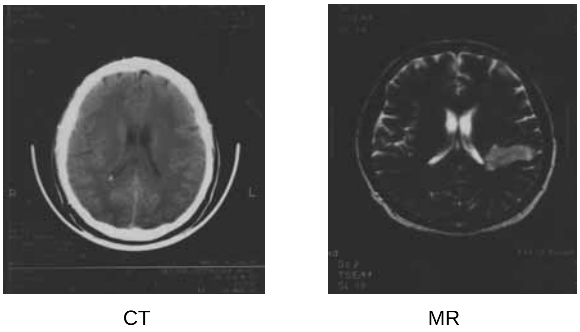

Fig. 1. Scan images.

SJ was initially screened for various cognitive functions. He was near perfect on the Bucco-Facial Apraxia task, where he was asked to perform facial motions, such as biting lips (14/15); his one error was on a word repetition trial. He had no trouble copying drawings of a clock and a flower (2/2). His lexical recognition and comprehension skills were on the whole remarkably intact: He scored 39/40 on auditory lexical discrimination, where he needed to decide whether two auditorially presented words sounded the same or not; 20/20 on the auditory word/visual word matching task, where an auditory word was to be matched to three visual words: the target, a semantic foil, and a phonological foil; 20/20 on an auditory lexical decision task, which included 10 words and 10 nonwords;[1] 50/50

[1] Note that here and throughout the method section, the nonwords are constructed by assembling two (or more) legal syllables together, e.g., /bei1ye4/ (cup-leaf). The *pinyin* symbols are used as the phonetic transcript in the article. The numbers following the phonetic transcripts of each syllable refer to the tone of the preceding syllable. There are four tones in Mandarin Chinese. Although different from nonwords in English or Italian, it is possible to read the nonwords we constructed simply by character-by-character reading, it does not mean that the patients necessarily do it that way. For instance, a patient of ours, ZBL (in preparation), displays symptoms of deep dyslexia and makes semantic errors in reading compounds as a whole and ignores the individual characters (e.g., read /you1 yu4/, depressed as /bei1 shang1/, sad), presumably because the lexical meaning of the whole word is driving the reading process. These patients are also likely to have more trouble reading the kind of nonwords we constructed than words.

on auditory word/picture matching, where an auditory word was to be matched to two pictures, including one semantic, visual, or phonological foil; 20/20 on an auditory sentence/picture-matching task, where two pictures were presented for an auditory sentence target; and 15/15 on visual word/picture matching, where a visual word was to be matched to four visual pictures. SJ was also reasonably good at written picture naming (10/11) and writing to dictation (9/10).

In contrast, his oral production capacity was severely impaired. He presented difficulty in oral repetition (24/40), oral picture naming (41/68), and oral word reading (26/57). We observed that he made many phonologically-related errors in oral tasks. The majority of errors were onset substitutions. For instance, he repeated */chang2bai2shan1/ as /pang2bai2shan1/*, */he1zui4jiu3/* as */he1sui4jiu3/*, read 喜欢 (*/xi3huan2/*) as */qi3huan1/*, 连贯 (*/lian2guan4/*) as */lian2gong4/*, and named the picture of a flower (*/hua1/*) as */shua1/*, of skipping (*/tiao4sheng2/*) as */diao4sheng2/*.

SJ was also given a comprehensive short-term memory test to evaluate whether his difficulty in the oral production tasks was due to some kind of short-term memory deficits where the activated information could not be retained before production. The tests covered digital span, syllable span, and object span. The syllable span further included one-syllable item lists; bisyllabic word lists; and two-syllable nonword lists. SJ was asked to repeat the list spoken by the experimenter. Since SJ had difficulties in oral production in general, the correct scores included the complete correct responses and those with sound similar to targets. In the object span test, the experimenter produced the names of a set of objects in front of SJ, and he was asked to point to the objects in the right sequence. Six normal adults were selected as SJ's control group. They were matched with SJ in sex (male), age (mean = 42.2, range = 40–50), and education level. SJ's short-term memory abilities appeared to be normal. In digit span tests, his forward digit span was 8 and his backward digit span was 3. They were comparable with the control group in both forward digit span (mean = 6.8, range = 6–8) and backward digit span (mean = 3.8, range = 3–5) tests. In three-syllable span tests, SJ presented normal memory capacity (5, 3, 3) compared to the controls (mean = 5.2, 3.3, 3.2; range = 4–6, 2–4, 2–4). His object memory span (6) was perfect, relative to control group (mean = 5.2, range = 4–6).

The results from the screening tests painted a general picture that SJ was intact in language comprehension and short-term memory and was impaired with respect to tasks involving oral production. To compare directly his performance across different modalities, we administered the following tests, where identical stimuli were used in different tasks and the sample size was greatly increased.

Table 1
Percentage of correct responses in modality tasks

| Task | Correct rate |
|---|---|
| Oral repetition | 56%(130/232) |
| Oral reading | 54%(125/232) |
| Oral picture naming | 62%(143/232) |
| Written picture naming | 91%(211/232) |
| Written to dictation | 91%(211/232) |
| Delayed copy | 94%(218/232) |

### 2.2. *Performance comparison across modalities*

#### 2.2.1. *Methods*

The material we used were 232 items (Set A) from Snodgrass & Vanderwart pictures [18]. These pictures were normed for Chinese speakers [17] on naming agreement, familiarity, imageability, and visual complexity. Six different tasks were administered on this set of stimuli: oral repetition, oral reading, oral picture naming, written picture naming, writing to dictation, and delayed copying. In the delayed copying task, each visual word was exposed for two seconds and then removed from sight. SJ was asked to write down the word from memory.

In the oral production tasks (repetition, reading, and picture naming), the 232 items were randomly divided into three blocks. Different blocks were used in oral repetition, oral reading, or oral picture naming in each testing session. SJ finished all three blocks in the three tasks within two weeks. Similarly, in the writing tasks, the items were divided into three blocks, and different blocks were used in the written picture naming, writing to dictation, or delayed copy task in each testing session. There was a two-week interval between oral and written tasks.

#### 2.2.2. *Results*

Table 1 lists SJ's correct rates on the tasks across modalities. Chi-square tests revealed no significant difference among the three oral tasks ($x^2(2) = 3.04, p = 0.22$). The majority of SJ's errors were phonologically-related errors (see the error analysis below).

By contrast, SJ carried out the written production tasks relatively well. No significant difference was found among the three written tasks ($x^2(2) = 1.90$, $p = 0.39$). On the whole, SJ completed written tasks

(93%) more accurately than oral tasks (58%) ($x^2(1) = 221.86, p < 0.0001$).

A post hoc analysis was conducted to discover whether word frequency (a lexical factor) and word length influenced SJ's difficulties in oral repetition and reading. The 232 Chinese words were split into 78 high-frequency (more than 10/million, Sun, et al. [20]), 79 medium-frequency (2–9/million), and 71 low-frequency (less than 1/million) groups. They were also divided into 36 one-character, 165 two-character, and 31 three- and four-character groups. The three frequency categories had no significant difference in repetition ($x^2(2) = 1.14$, $p = 0.57$) and in reading ($x^2(2) = 0$). However, we observed a length effect in these two tasks (repetition, $x^2(2) = 9.70$, $p < 0.01$; reading, $x^2(2) = 21.37, p < 0.0001$). His correct percentage for repeating one-, two-, and three- and four-character words were 78%, 54%, and 42%, respectively, and those for reading were 67%, 58%, and 16%, respectively.

In summary, we replicated here the preliminary findings with a within-item design and a greater N and confirmed that SJ had an equivalent degree of impairment in oral tasks. We also observed a trend in the post hoc analyses: His oral production performance was affected by word length and not by word frequency.

To understand better which component impairment was responsible for SJ's difficulty in the oral production task, it was important to reveal which factors of the stimuli contributed to his performance. If his impairment was located at the (higher) levels of semantic and/or lexical representations, his performance should be a function of conceptual/lexical factors such as concreteness, frequency, and grammatical class. An impairment to a lower level, such as an output buffer, should show patterns that are sensitive to the capacity of the buffer, namely, the word length. Because the Snodgrass & Vanderwart items were not designed for examining various lexical factors, and the items in different frequency categories were not matched on other factors such as word length, the following experimental session was specifically constructed to address this issue.

### 2.3. Investigating the effects of lexical factors

#### 2.3.1. Methods

The following sets of stimuli were constructed to examine the effect of relevant lexical variables on SJ's performance.

*Word frequency effect and concreteness effect:* Material Set B included 80 two-character words, with 40 high frequency (> 50/million) and 40 low frequency (< 10/million) words [20]. All of the words could be further separated into abstract and concrete words. So there were 20 high-frequency and 20 low-frequency abstract words and 20 high-frequency and 20 low-frequency concrete words. Concreteness was rated by 20 college students on a 5-point scale, with 1 being most abstract and 5 being most concrete. The mean score of abstract words was 1.2, and the mean rating of concrete words was 3.9.

*Word frequency effect and grammatical class effect:* Set C included 240 two-character words with different word classes to probe any potential grammatical class effect. It consisted of 40 high- and 40 low-frequency nouns (120/million and 2/million, respectively), 40 high- and 40 low-frequency verbs (114/million and 3/million, respectively) and 40 high- and 40 low-frequency adjectives (109/million and 4/million, respectively).

*Lexicality effect:* Set D contained 330 two-syllable words, including half real words and half nonwords. The 165 real words were derived from all two-character words in Chinese names [17] of Snodgrass & Vanderwart pictures [18]. The nonwords were created by the following procedure. We reversed the first and second characters of 165 real words, and then one from the first character set and one from the second character set were randomly paired.

*Word length effect:* Set E had 160 nouns, with 40 one-character, 40 two-character, 40 three-character, and 40 four-character words. The word frequency was matched among four groups (2.7/million, 4.0/million, 3.2/million, and 2.8/million, respectively).

#### 2.3.2. Results

SJ's performance in the various tests is listed in Table 2. We did not find any frequency effect in oral repetition and reading tasks Set B, repetition: $x^2(1) = 1.73$, $p = 0.19$; reading: $x^2(1) < 1$; Set C, repetition: $x^2(1) = 1.96, p = 0.16$; reading: $x^2(1) < 1$). There was no indication of any concreteness effect in repetition or reading ($x^2(1) < 1$). No effect of grammatical class was observed either. The correct percentage were comparable for nouns, verbs, and adjectives in Set C: repetition ($x^2(2) < 1$) and reading ($x^2(2) < 1$). Furthermore, we did not find any word/nonword differences in Set D: repetition or reading ($x^2(1) < 1$). However, we did find a significant word length effect in Set E: repetition, $x^2(3) = 23.80, p < 0.0001$; reading, $x^2(3) = 10.21, p < 0.05$.

Table 2
Correct percentage of various effects in oral production tasks

| Examined effect | | Task | |
|---|---|---|---|
| | | Repetition | Reading |
| *Word frequency* | | | |
| Set B | High | 83%(33/40) | 65%(26/40) |
| | Low | 70%(28/40) | 75%(30/40) |
| Set C | High | 82%(98/120) | 72%(86/120) |
| | Low | 74%(89/120) | 73%(87/120) |
| *Concreteness effect* | | | |
| Set B | Concrete | 80%(32/40) | 70%(28/40) |
| | Abstract | 73%(29/40) | 70%(28/40) |
| *Word class effect* | | | |
| Set C | Noun | 76%(61/80) | 70%(56/80) |
| | Verb | 76%(61/80) | 70%(56/80) |
| | Adjective | 81%(65/80) | 75%(60/80) |
| *Lexicality effect* | | | |
| Set D | Real word | 73%(121/165) | 79%(130/165) |
| | Nonword | 73%(121/165) | 75%(124/165) |
| *Word length* | | | |
| Set E | One-character word | 93%(37/40) | 88%(35/40) |
| | Two-character word | 70%(28/40) | 75%(30/40) |
| | Three-character word | 58%(23/40) | 60%(24/40) |
| | Four-character word | 43%(17/40) | 60%(24/40) |

### 2.4. Error analysis

We first attempted to code the errors into six main types: 1) Semantic-related errors mean that the error response (e.g., apple) was semantically related to the target item (orange); 2) Phonologically similar errors mean that the error response was phonologically related to the target item; 3) Mixed errors refer to the error response (e.g., mao2, cat) that was not only semantically related but also phonological related to the target item (e.g., bao4, leopard); 4) Visual errors mean that, in picture-naming tasks, the target item (e.g., toothbrush) was named as a visually similar object (e.g., pen); 5) Unrelated errors; 6) No response errors.

We analyzed SJ's performance in 232 Snodgrass & Vanderwart items (Set A) and discovered that all of his errors in all tasks were phonological errors, except for one "no response" error in picture naming (cherry). The number of errors on oral repetition, reading, and picture-naming tasks were 102, 107, and 89, respectively.

To elucidate the distribution characteristics of phonological errors in oral production, we further collected all the phonological errors across different stimuli sets. There were 828 words with 1,846 syllables in the oral repetition and reading tasks, and 232 words with 461 syllables in the oral picture-naming task. In each task, we first calculated the number of erroneous syllables. Then we broke down the phonological errors into onset, rime, tone errors, and possible combinations. The onset errors were defined as those where the response (e.g., */jiu2/*) was different from the target item (e.g., */niu2/*) in the onset. The rime errors and tone errors were those responses different in the rime (e.g., */hua1/-> /huan1/*) and in the tone (e.g., */lian4 -> lian3/*), respectively. The results (see Table 3) demonstrate that most of the phonological errors are single errors, meaning that the patient made mistakes only on the onset (e.g., */li3mao4/ -> /li3bao4/*), rime (e.g., */bao4en1/ -> /bo4en1/*) or tone (e.g., */gan3dong4/ -> /gan1dong4/*). In all of the three tasks, the most common type of single error was an onset error.

We further separated SJ's main error type, onset errors, into substitution, insertion, deletion, and anticipation/perseveration. Substitution error means an error in which an onset phoneme (e.g., */mao4/*) was replaced by another onset phoneme (e.g., */bao4/*). Insertion error means an error in which an onset phoneme (e.g., */ch-she4/*) was mistakenly added into the target (e.g., */she4/*). Deletion error means an error in which an onset phoneme was lost. Anticipation error means an error in which the onset phoneme in the first syllable was replaced by the onset phoneme of the second syllable within a compound word (e.g., */qi4che1/ -> /che4che1/*); perseverations refer to those errors where the onset phoneme of the second syllable was replaced by the onset of the first (e.g., */di4tu2/ -> /di4du2/*). Table 4 shows that onset substitution error is the main

Table 3
Distribution of various subtypes of phonological similar errors in oral production tasks

| Error type | Repetition ($N = 246$) | Reading ($N = 240$) | Picture naming ($N = 29$) |
|---|---|---|---|
| Onset | 80% | 69% | 72% |
| Rime | 14% | 16% | 14% |
| Tone | 1% | 1% | 7% |
| Onset + rime | 4% | 8% | 7% |
| Onse + tone | 1% | 1% | 0% |
| Rime + tone | 0% | 0% | 0% |
| Onset + rime + tone | 0% | 4% | 0% |
| Total | 100% | 100% | 100% |

Table 4
Distribution of various subtypes of onset errors in oral production tasks

| Error type | Repetition ($N = 194$) | Reading ($N = 165$) | Picture naming ($N = 21$) | Total ($N = 380$) |
|---|---|---|---|---|
| Substitution | 72% | 70% | 71% | 71% |
| Insertion | 24% | 22% | 24% | 23% |
| Deletion | 0% | 0% | 0% | 0% |
| Anticipation/ perseveration | 4% | 8% | 5% | 6% |

type of error in all of the tasks, and its occurrences in the three tasks are comparable ($x^2(2) < 1$).

When an onset was mistakenly substituted by another onset, what drove the production of the erroneous onset? Table 5 displays all of the target onsets and the instances that certain erroneous onsets were produced in SJ's 645 errors of onset substitutions in reading, repetition, and picture-naming tasks. The schematic presentation considered the features of the onsets of modern standard Chinese, generally grouping the onsets into four major articulators: labial (*b, p, m, f, w*), coronal (*d, t n, l, z ,c ,s, zh, ch, sh, r*), dorsal (*g, k, h, zero*), and ambiguous (*j, q, x, y*) [10]. The onsets within each category were listed grouped by the subordinate features-continuant, occlusive, etc. [10]. Analyses reveal that the substitution errors produced by SJ had 27 within-labial errors (labial onsets replaced by another labial onset), 222 within-coronal errors, and 41 within-dorsal errors.

Efforts were further made to compare the within-class substitution rates to the random baselines. The random baselines were calculated by dividing the total number of onset by the total number of the particular kind of onset. Take labial onsets as an example. Among the 24 Chinese onsets, there are five labial ones. Thus, the number of within-labial substitution errors in 645 target onsets by chance was calculated using this formula: 645 $\times$ (5/24) $\times$ (5/24) = 21. And then, we examined whether the observed value (27/645) was significantly higher than the expected value by chance (21/645). We found that the within-category substitution rate was not higher than chance for the labial category ($x^2(1) = 0.87, p = 0.38$), but was for the coronal (222/645 vs. 135/645, $x^2(1) = 29.31$, $p < 0.001$) and the dorsal (47/645 vs. 18/645, $x^2(1) = 13.63$, $p < 0.001$) categories.

## 3. Discussion

We reported a patient, SJ, who has normal comprehension ability but is impaired with all tasks involving oral production, such as reading, repeating, and oral naming of words and nonwords. More informative are the types of errors he makes, which are phonological in nature and of the same pattern across the different oral production tasks. We briefly summarize the features of his performance here.

1. In the reading, oral naming, and repetition of words, he produces common patterns of errors in terms of the percentage of error and the distribution of various types of errors.
2. The likelihood of making errors is significantly affected by the length of the stimuli, but not the concreteness, frequency, or grammatical class.
3. The errors are exclusively phonological in nature, the majority of which are onset substitutions, with the rimes and tones being relatively spared. Rimes and tones are never mistaken together.
4. The performance on reading and repetition of nonwords is similar to that of real words in every aspect.

Table 5
Matrix of onset substitution errors in oral production tasks (the target onsets by rows and the response onsets by columns)

| | | Labial | | | | | Coronal | | | | | | | | | | | Dorsal | | | | Ambiguous | | | |
|---|---|---|---|---|---|---|---|---|---|---|---|---|---|---|---|---|---|---|---|---|---|---|---|---|---|
| | | b | p | m | f | w | d | t | n | l | z | c | s | zh | ch | sh | r | g | k | h | zero | j | q | x | y |
| Labial | b | | 1 | 2 | | 1 | 2 | | | | | | | | | | | | | | | | | 1 | |
| | p | 6 | | | | | | 1 | | | | | | | | 1 | | | 1 | | | | 1 | 1 | |
| | m | 8 | | | | 2 | | | 1 | 2 | | | | | | | | | | | | | | 2 | |
| | f | | 1 | | | 1 | | | | | 1 | 3 | | | 3 | 1 | | | 1 | 5 | | | | | |
| | w | | | 3 | 2 | | | | | 2 | | | | | | | | 1 | | 2 | | | | 1 | |
| Coronal | d | 4 | | | | | | 2 | 1 | 1 | 1 | | | 5 | 1 | 2 | | 2 | | | | 2 | 1 | 1 | 2 |
| | t | | 2 | | | | | | | 2 | | 1 | 2 | | 8 | 5 | | | 3 | | | 2 | 2 | | |
| | n | | | 3 | | | | | | 3 | | | | | | 1 | | | | 1 | | 1 | | | 1 |
| | l | 1 | | 2 | | | 3 | | 8 | | | | | | 1 | | 2 | 2 | | 2 | | | | 1 | 9 |
| | z | | | | | | | | | | | 4 | 2 | 7 | 2 | 2 | | | | | | | 1 | 1 | |
| | c | | | | | | | | | | 1 | | 5 | | 11 | | | | 1 | 1 | | | | 1 | |
| | s | | | | 2 | | | | | | 1 | 11 | | | 3 | 12 | | | | 1 | | | | | |
| | zh | | | | | | 4 | | | 1 | 8 | | | | 3 | | | 3 | | | | 5 | | | |
| | ch | | | | 6 | | | 2 | | | | 6 | 3 | 4 | | 12 | 1 | | 1 | 1 | | | 4 | | |
| | sh | | 1 | | 4 | | | 1 | | | | | 11 | 2 | 48 | | | | | 10 | | | 2 | 5 | 1 |
| | r | | | | | 1 | | | | 5 | | | | | | 1 | | | | | | | | | |
| Dorsal | g | | | | | 1 | 1 | | | | 1 | 2 | | 3 | | 1 | | | 9 | 6 | | 2 | 1 | 1 | 1 |
| | k | | | | 1 | | 1 | 1 | | | | 1 | 1 | | 4 | 3 | | | | 14 | | | 1 | 1 | |
| | h | 1 | | | 7 | | | | | | | 1 | 1 | 1 | 10 | 12 | | 1 | 11 | | | | 2 | 1 | |
| | zero | | | | | | | | | | | | | | | | | | | | | | | | |

5. Word writing (writing to dictation, written picture naming) is relatively spared.

What kind of deficit best explains SJ's performance? The selective impairment of the phonological output buffer accounts for the impairments in all oral production tasks naturally. First of all, the phonological output buffer is likely to be the common cognitive component in these oral production tasks [5,15]. Second, because the phonological output buffer is a stage that comes after the semantic and lexical selection, the effected semantic and lexical factors are not predicted; being a buffer, the structure should be sensitive to the amount of the information (length), given that presumably the capacity is limited; the phonological output buffer holds the phonological representation, which explains the phonological nature of the errors. Furthermore, there was no indication that SJ had impaired short-term memory. His difficulty with the phonological output buffer, in spite of having a significant length effect, is not to be explained by degradation in short-term memory in general.[2]

We believe that our patient's behaviors provide information about both the structural and functional aspects of the phonological output buffer in language production processing. The issue of the structural characteristics of the phonological output buffer concerns the fashion in which the phonological output buffer retains phonological information. SJ tended to make more errors with the onsets of syllables than with the rimes and tones. The rimes and tones were never mistaken together. Therefore, the phonological output buffer for Chinese speakers has to be structured in a way that allows the onset, rimes, and tones to be impaired independently, for the onset to be more impaired than other parts of the syllables and tones. Within the onset errors, the articulator-based feature dimension we looked at seemed to have an effect such that the articulator-based categorical boundaries (coronal and dorsal) were respected in the substitution errors.

What information about the functional status of the phonological output buffer can we infer from our patient's patterns? The performance patterns that are associated with the selective phonological buffer deficit presented by previously reported cases [5,15] are as

[2] A possible alternative explanation of SJ's impairments should be considered: Instead of "phonetic substitution," SJ's errors are actually "phonemic distortation" due to impairments to some fine articulatary programs. In other words, the locus of the deficit might be more peripheral that the phonological output buffer, and when he cannot employ the correct articulatory program to produce the target onset, he tends to output another sound that employs a similar/easier articulatory program. This explanation, however, is not likely the case because when he made an error on a target onset, the erroneous onset produced quite often did not involve an easier or similar articulatory program (see Table 5). Also, this account does not obviously predict the significant length effect exhibited by SJ. We thank anonymous reviewer 1, Alfonso Caramazza, and Zhiqiang Li for discussing this alternative with us.

follows: 1) the error patterns in reading, repetition, and writing were comparable in every aspect; 2) The error patterns in reading, repetition, and writing had a significant length effect; 3) Errors were predominantly single phoneme errors that bear phonological relationships with target phonemes.

Comparing these with the five features of SJ's performance we characterized earlier, we see that SJ is similar to the other phonological output buffer patients, especially in terms of the nature of his errors. It is also worth noting that, consistent with other phonological output buffer patients, SJ's comprehension is preserved, which is consistent with the hypothesis that separate buffer systems underlie the input and output processes [12,15]. Yet, SJ differs from the previous four cases in two aspects: the perfect association between word and nonword processing and the sparing of writing.

Different from the previously reported phonological buffer deficit cases, the word processing and nonword processing were affected to the same degree in our patient, SJ. Patients IGR, MV, and RR showed impairment only in nonword processing. LT's impairment with respect to nonwords and words were comparable qualitatively, but not quantitatively. This difference between SJ and the other patients might be trivial. As we pointed out earlier, the nonwords in our tests were constructed by combining legal characters/syllables, and it can be argued that they are real lexical items on the character/syllable level and can be processed just as lexical words once they are decomposed in the reading and repetition tasks. In English and other alphabetic languages such as Italian, however, the nonword tasks cannot be accomplished this way but only through applying grapheme-phoneme conversion rules.

Another significant difference was that SJ's deficit was restricted to the oral production modality, and the written modality of words was relatively spared. Because nonword writing cannot be tested in Chinese, SJ is not directly comparable to IGR and RR, whose reading and repetition deficit with nonwords were associated with nonword writing. LT, however, did show the same kind of impairment in word writing as in word reading and repetition. We believe that this difference between SJ and LT might reflect the difference in nature of specific languages.

As presented in the Introduction, it is consistent in the functional architecture of the writing process that nonword writing goes through the phonological output buffer. The accepted view of writing (e.g. [21]) entails a lexical route (the target lexical orthographic node activates its own graphemic representation) and a nonlexical route where the phonological output buffer holds the phonological contents that will be converted into graphemic nodes through sound-to-grapheme conversion rules. Although it is still unclear how the information from these two routes integrates (e.g. [11]), various kinds of empirical evidence support the existence of these two routes [4,21].

If the only locus of a patient's deficit is the phonological output buffer, it does not follow from this architecture of writing that the patient will show the same pattern of errors in writing as in other oral production modalities, given that in writing the intact lexical route also generates graphemic representations. Even if the nonlexical route outputs the incorrect graphemic representation from the impoverished phonological output buffer, the effect from the lexical route should either compensate for the errors or modulate the types of erroneous output. The association between word oral productions (reading and repetition) and written production should not be a signature pattern of the phonological output buffer deficit.

In the literature, among those who were classified as selective phonological output buffer deficit patients, LT is the only one whose word processing impairment was visible. His performance on the word writing-to-dictation task was not different from the word reading and repetition performance. We discussed in the Introduction three possible accounts for this association. The first was that it is accidental that LT is also impaired along the lexical route of writing. Second, Italian is a highly transparent language and, hence, the nonlexical route is heavily relied on in writing to dictation, masking the integration from the lexical route. Both of these hypotheses explain the absence of such an association in our patient, SJ, because the nonlexical route of writing is virtually absent in Chinese. The third hypothesis assumes that the phonological output buffer is involved even in the lexical route of writing by participating in some "refreshing mechanism" of the graphemic output buffer–but this cannot accommodate the sparing of writing words in SJ.

To conclude, the impairment of the Chinese-speaking patient we documented in this paper is best explained by the selective impairment of the phonological output buffer. His erroneous patterns were both qualitatively and quantitatively equivalent across reading and repetition for both words and nonwords and the oral naming of words. The general patterns revealed by the error analyses are similar to other English- and Italian-speaking patients who are classified as having the same locus of impairment. However, SJ's difficulty in oral production is not accompanied by the same kind of difficulty in written production tasks. This finding is consistent with the classical view of writing, which assumes that there is a lexical route bypassing the phonological output buffer. It clearly demonstrates that the association between the impairment of writing and oral production is not necessarily predicted by the phonological output buffer deficit, although it has been seen in other patients of the same type. Furthermore, SJ's performance also reveals that the onset, the rime, and the tone of a syllable are represented independently in the buffer.

## Acknowledgements

This research was supported by the Natural Science Foundation of China (#30070259 and #60033020), the National Pandeng Project (95-special-09), and a grant from Key Laboratory of Applied Psychology, Beijing. Please address correspondence to: Hua Shu, State Key Laboratory of Cognitive Neuroscience and Learning, School of Psychology, Beijing Normal University, No. 19, Xin Jie Kou Wai Street, Beijing, 100875, China. E-mail: shuh@bnu.edu.cn.
